# Highly Flowable Nano TiO_2_/Porous Organic Polymer (POP) Supports for Efficient Metallocene Catalysts

**DOI:** 10.3390/nano11010060

**Published:** 2020-12-29

**Authors:** Xiong Wang, Wenqian Kang, Lin Gao, Guangquan Li, Xuerong Chen, Yi Guo

**Affiliations:** Lanzhou Petrochemical Research Center, Petrochemical Research Institute, PetroChina, Lanzhou 730060, China; kangwenqian@petrochina.com.cn (W.K.); gaolin1@petrochina.com.cn (L.G.); liguangquan@petrochina.com.cn (G.L.); chenxuerong@petrochin.com.cn (X.C.); guoyi2@petrochina.com.cn (Y.G.)

**Keywords:** nano titanium oxide, porous organic polymers (POPs), metallocene catalyst, ethylene polymerization

## Abstract

Porous organic polymers (POPs) have proven to be an efficient support in the olefin polymerization catalyst field. In this paper, nano TiO_2_ beads were used to modulate the pore structure, bulk density, and surface morphology and flowability of the prepared POPs. With the incorporation of the hydrophilic nano TiO_2_ beads, the prepared TiO_2_/POP supports obtained reasonable specific surface area (100–300 m^2^/g) and higher bulk density (0.26–0.35 g/mL) and flowability than the pure POP supports. The results show that bulk density of the prepared TiO_2_/POP particles increased when adding an increased amount of TiO_2_, and when 37.5% TiO_2_ (weight percent to the total comonomers divinylbenzene (DVB) and 2-hydroxyethyl methacrylate (HEMA)) and 3:1 DVB/HEMA (molar ratio) were added, highly flowable TiO_2_/POP composites (POP-6 and POP-7) were obtained. With the modulation of the nano TiO_2_ template during the support synthesis, the prepared POP-7 particles successfully achieved a normal distribution with a narrow particle size distribution (PSD) of 0.717 and average particle size of 24.1 μm, a specific surface area (SSA) of 279 m^2^/g, and relatively high bulk density of 0.30 g/mL. Furthermore, all the prepared TiO_2_/POP supports obtained higher ethylene polymerization activity than silica gel-supported commercial metallocene catalyst. The immobilized (n-BuCp)_2_ZrCl_2_/MAO@POP-7 catalyst exhibited the highest ethylene polymerization activity of 4794 kg PE/mol Zr.bar.h and productivity of 389 g PE/g cat, more than twice that of the commercial counterpart. Even higher catalyst productivity (3197 g PE/g cat) and bulk density of the produced PE (0.36 g/mL) could be obtained in higher ethylene partial pressure at 80 °C for 2 h, and the prepared TiO_2_/POP catalyst shows no obvious Zr^+^ active sites decay during the ethylene polymerization.

## 1. Introduction

Polyolefins are undoubtedly one of the most robust fields in polymer production and consumption globally [1,2,3]. Along with continuously growing demand worldwide, there is the remarkable progress of polyolefin catalysts and polymerization process technologies [4,5,6,7,8,9,10]. The single-site metallocene catalysts have attracted extensive interest, both from academia and industry, due to their abilities to precisely control the polymer microstructure and stereospecificity by simply tailoring the ligands used [11,12,13,14,15]. 

Heterogeneous catalysis is required in the gas-phase or the slurry process for industrial applications to avoid reactor fouling and to control the polymer morphology or the forming granule reactor by immobilization of the homogeneous catalytic sites into a carrier [16,17,18,19,20]. Inorganic supports, such as silica gel, magnesium chloride, aluminum oxide, zeolite, molecular sieves, etc., have been reported as metallocene catalysts carriers in numerous literatures. Some of the inorganic supports used as metallocene catalysts were successfully commercialized. However, they often need fastidious treatment to remove the surface acidic groups, which leads to active sites deactivation of the supported metallocene catalysts, and the inorganic residual may affect the produced polymer properties [21,22,23,24]. 

The porous organic polymers (POPs) are a potential alternative for commercial immobilized metallocene catalysts, and they have attracted ever-increasing attention in a wide range of applications for the last two decades due to their unique properties, such as high surface area, varied synthesis strategy, easy functionality, excellent thermal stability, etc. [25,26,27,28,29,30]. They also provide a more homogeneous environment for active sites during the olefin polymerization, and they need no complex pre-treatment for immobilization and suffer from no severe catalytic deactivation due to the surface acidic group. A great deal of POPs with different functional groups were prepared for polyolefin catalysts support, and they exhibited excellent ethylene polymerization activity. We designed and synthesized a series of POPs with different functional comonomers, such as HEMA, 2-hydroxypropyl methacrylate (HPMA), and 4-vinylbenzyl chloride (VBC), and we also discussed the feasibility for synthesis of different pore structure, bulk density, and morphology via a dispersion polymerization method, and the highly porous polymers showed excellent catalytic performance [31,32,33,34].

However, the particle size, bulk density, and surface morphology of the prepared POPs are hard to control and generally have bad flowability as a catalysts support, due to the thermodynamic incompatibility between solvent, comonomers, and the prepared polymers, which limited their commercial applications as catalyst support. A small number of nano metal oxides [32], such as Fe_3_O_4_, Fe_2_O_3_, and CuO, were introduced in the preparation of POPs to tune pore structure, bulk density, morphology, etc., and then the added metal oxides were removed by acid etching for olefin polymerization catalysts.

In this paper, nano TiO_2_ beads were selected for an effective template agent to modulate the pore structure and particle morphology of the prepared TiO_2_/POP composites without needing to remove the metal oxide, and highly free-flowing TiO_2_/POP composites with higher bulk density, narrow particle size distribution, and high ethylene catalytic activity were obtained.

## 2. Experimental Section

### 2.1. Materials

Divinylbenzene (DVB, 55% and 80%, Aladinn Reagent, Shanghai, China), 2-hydroxyethylmethacrylate (HEMA), and 2,2-Azo-bis-isobutyronitrile (AIBN, A.R. Aladdin Reagent, Shanghai, China) were treated according to our previous work before use [31,32]. Ethanol (99.5%, Tianjing Yongda Chemical Co., Tianjing, China), POE-b-POP (Poloxamer 407, F127, BASF), deionized water (provided by Lanzhou Petrochemical Center, Lanzhou, China), and titanium (IV) oxide (≥99.8% metals basis, 100 nm beads, anatase, hydrophilic, Aladinn Reagent, Shanghai, China) were used as received. Methylaluminoxane (MAO) (10% in toluene, purchased from Albermarle, which is now known as GRACE) was vacuum distilled to remove toluene and the residual trimethylaluminum (TMA) and obtain white MAO powder. Bis (n-butylcyclopentadienyl) zirconium dichloride ((n-BuCp_2_)ZrCl_2_) (≥98%, DAL CHEM, Nizhny Novgorod, Russia) was directly used without further treatment. 

### 2.2. Preparation of TiO_2_/POP Supports

The TiO_2_/POP composites were synthesized by free radical polymerization using a similar method adopted by our group [31,32]. Typically, a certain amount of hydrophilic nano TiO_2_ beads were added in 120 mL Ethanol and deionized water mixture solvent (*V*:*V* = 9:1) in a multi-necked glass reactor. They were then evenly-dispersed in solvent with ultrasonication for 3 min. At a stirring speed of 360 rpm, 4.80 g of DVB, 1.60 g of HEMA, and 0.128 g F127 (2 wt%) were added into the reactor. Then they were dissolved in the solvent by heating to 50 °C for 1 h. Then 0.128 g (2 wt%) AIBN was added into the reactor to initiate a radical polymerization reaction at 70 °C for 3 h and aging for another 5 h. After polymerization, the TiO_2_/POP composites were filtered at 70 °C, washed with hot ethanol or an ethanol/water mixture solvent and vacuum filtered three or four times to remove impurities. To complete the process, the prepared white or off-white TiO_2_/POP composites were vacuum treated at 70 °C for 8 h for further use.

### 2.3. Immobilization of Metallocene Catalysts

To begin, 2.84 g TiO_2_/POP support and 1.56 g MAO white powder were added in 80 mL dry toluene and stirred at ambient temperature for 1h. Then 0.114 g (n-BuCp)_2_ZrCl_2_ complex was added while stirring for another 2 h. The prepared light-yellow solids were washed in toluene and hexane three times, respectively, and were then were dried under vacuum to obtain free-flowing metallocene catalyst particles. The metallocene catalysts immobilized in TiO_2_/POPs were ready for use in ethylene homopolymerization.

### 2.4. Ethylene Polymerization

Ethylene homopolymerization was carried out in an 800 mL stainless steel reactor with an external oil bath for temperature control. 300 mL hexane solvent and 3 mL TEAL (10% in hexane) were added in the pressure reactor after high-purity nitrogen purging. After stirring for 5 min, about 130 mg of the prepared (n-BuCp)_2_ZrCl_2_/MAO@TiO_2_/POP catalyst was added. The polymerization was conducted at 80 °C and 4 bar ethylene pressure for 30 min. The ethylene pressure was kept constant during the whole process of ethylene polymerization. Ethylene polymerization was also conducted in a 10 L pressure reactor for polymerization kinetics at 80 °C.

### 2.5. Characterization

The pore structure of the TiO_2_/POPs support was evaluated on a Nova 2000e Nitrogen sorption porosimetry (Quantachrome Instruments, Boynton Beach, FL, USA). Before testing, these samples were vacuum-dried at 120 °C for 8 h to remove residuals on the surface, and then the testing was conducted at the temperature of liquid nitrogen (77.3 K). Particle size and particle size distribution were carried out on a Mastersizer 2000 (Malvern, Malvern city, UK) using ethanol as dispersion medium. The IR analysis was conducted on a NEXUS 670 FTIR (Glendale, WI, USA). A DSC Q2000 (New Castle, DE, USA) was used for thermogravimetric (TG) analysis in nitrogen protection with 20 °C heating rate from room temperature to 800 °C. X-ray diffraction (XRD) testing was performed on a Bruker D8 ADVANCE (Bruker, Karsruher, Germany) using Cu Kα radiation (λ = 1.5406 Å) with 2θ scanning angle from 10° to 70°. A scanning electron microscope (SEM) was used on a HITACHI S4800 (Tokyo, Japan) to analyze the surface morphology of the prepared support. The support particles were dispersed on electric glue mounted on a metallic base. Then the samples were sprayed with a thin layer of gold before the test. Element analysis of the metallocene catalysts was carried out on a VISTA ICP-MPX (VARIAN, Palo Alto, CA, USA) to obtain Al and Zr loading according to the literature [33].

## 3. Results and Discussion

### 3.1. Preparation of Nano TiO_2_/POP Particles 

In this work, the nano TiO_2_ beads were used as a significant component to modulate the bulk density, surface morphology, and flowability of the produced POP particles. The TiO_2_/POP particles were prepared by a dispersion or precipitation method, and the pore structure parameters and the bulk density of the inorganic/organic composite supports are listed in Table 1. As seen in Table 1, the TiO_2_/POP particles obtained a variety of porous structures with specific surface area (SSA) from about 100 to 350 m^2^/g, and a pore volume from about 0.15 to 0.40 mL/g, which is suitable for a potential metallocene catalysts support. Moreover, the prepared free-flow TiO_2_/POP particles gained relatively higher bulk density of 0.25–0.35 g/mL, compared to the previous method [31,32,33]. 

**Figure 1:**
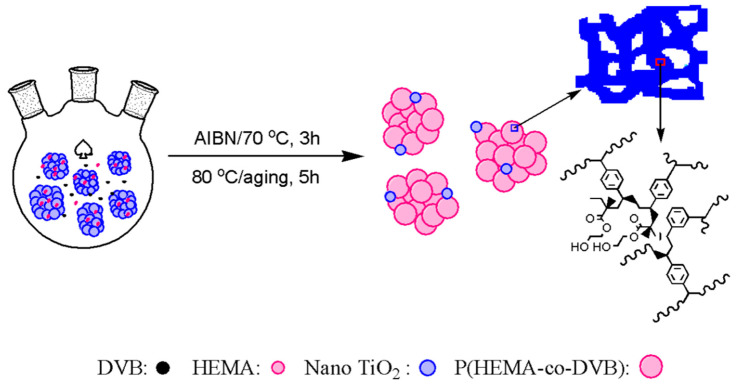
Preparation of TiO_2_/POP particles.

Similar with the particle-forming mechanism of POPs by a metal oxide template, the hydrophilic TiO_2_ nano-aggregates could absorb functional monomers and play as a template during the radical polymerization. As illustrated in Figure 1, the POP particles grow around the nano TiO_2_ beads or nano aggregates. As the POPs grow larger around the inorganic particles, the nano TiO_2_ particles could be dispersed in the prepared POP matrix in order to regulate the pore structure, bulk density, and flowability of the TiO_2_/POP particles. Based on this mechanism, highly flowable TiO_2_/POP particles were prepared (See supplementary video clip).

### 3.2. Pore Structure of the Prepared TiO_2_/POP Particles

**Figure 2:**
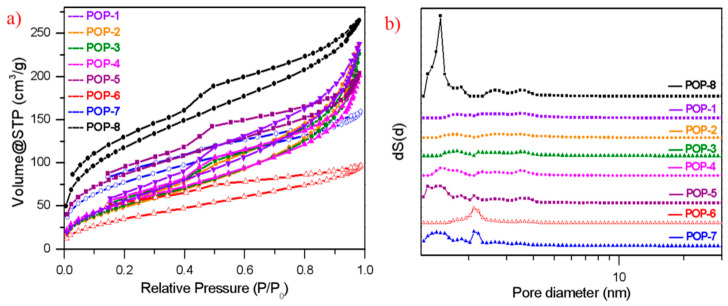
(**a**) N_2_ isotherms, (**b**) non-local density functional theory (NLDFT) pore size distribution curves of the prepared TiO_2_/POPs.

As seen from Table 1 and Figure 2, similar pore structure was obtained with the same comonomers formula and cross-linking degree of DVB. When 55% cross-linking degree DVB and a DVB/HEMA molar ratio of 3:1 were added, the pore structure of the prepared TiO_2_/POP particles (POP 1–4) varied a little with similar nitrogen isotherm and pore size distribution curves. The specific surface area and pore volume of the four samples were about 190–220 m^2^/g and 0.31–0.37 cm^3^/g, respectively. Compared to our previous work, the bulk density of the prepared P(DVB-co-HEMA) supports typically ranges from 0.15 to 0.30 g/mL. However, higher bulk density of the prepared TiO_2_/POP composites can be achieved by adding nano-TiO_2_, with bulk density ranging from 0.26 to 0.35 g/mL, which approved that the nano-TiO_2_ is an effective component to tune the bulk density of the prepared supports. 

The cross-linking degree of DVB and the functional comonomer HEMA are also significant pore-modulating factors of the prepared TiO_2_/POP particles. Typically, when higher cross-linking DVB was used, more highly porous TiO_2_/POP particles can be obtained due to higher cross-linking degree in the produced polymer network. The characteristic pore size peak of around 1.475 nm due to a higher cross-linking of DVB could be clearly observed in POP-5, POP-7, and POP-8 (see Figure 2b). The SSA of POP-5 was increased to 309 m^2^/g from 204 m^2^/g of POP-3, and POP -7 had a higher SSA of 279 m^2^/g compared to 136 m^2^/g of POP-6. The SSA of POP-6 decreased from about 200 m^2^/g to 136 m^2^/g when decreasing the HEMA content from 3:2 to 3:1 of DVB/HEMA molar ratio. An obvious peak around 2.21 nm of POP-6 and POP-7 could be noticed in the curves of NLDFT pore size distribution, and the decrease in HEMA content also resulted in the less porous N_2_ isotherm in consequence of the reduced pore size distributions mainly from 2.3 to 5.0 nm. The reason why the HEMA content influences the pore size distribution was discussed in detail in our previous research [33]; it is mainly caused by the change in solubility parameter of the prepared polymer when increasing the HEMA content.

### 3.3. IR and TGA Analysis

**Figure 3:**
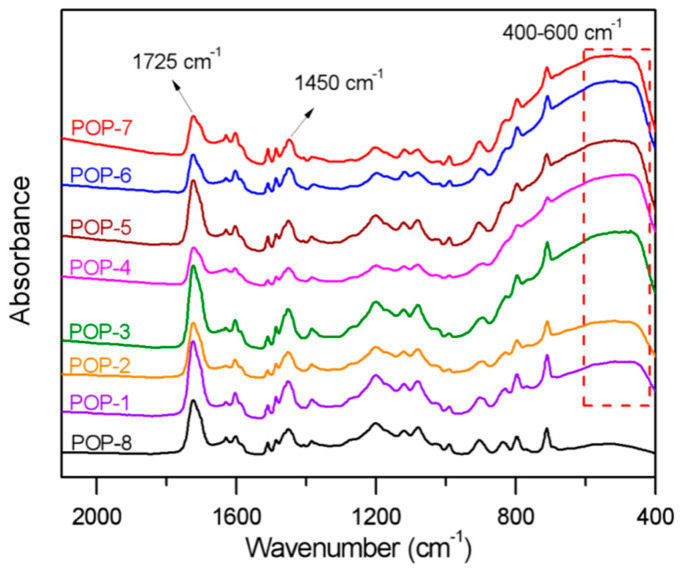
IR spectra of the prepared TiO_2_/POP particles.

The IR spectra of the prepared TiO_2_/POP particles are shown in Figure 3. The characteristic peaks of C=O in the HEMA units can be clearly observed around 1725 cm^−1^, and the bands around 1450 cm^−1^ of the in-plane bending mode of $\tilde{\upsilon }$(C-H) of -CH_2_- also exist in all samples. There is little absorbance in the P(DVB-co-HEMA) of POP-8, while as a comparison, there are unambiguously broad absorbance bands of around 400–600 cm^−1^ in the prepared TiO_2_/POP composites, which can be ascribed to the characteristic peak of TiO_2_. Moreover, the HEMA unit and TiO_2_ relatively content in the TiO_2_/POP composites can be evaluated by the specific value of the C=O and TiO_2_ characteristic peak height (area) to the calibrated peak height (area) in 1450 cm^−1^. As a result, the HEMA unit and TiO_2_ relatively content in the TiO_2_/POP composites are in accordance with the added content of HEMA and TiO_2_.

**Figure 4:**
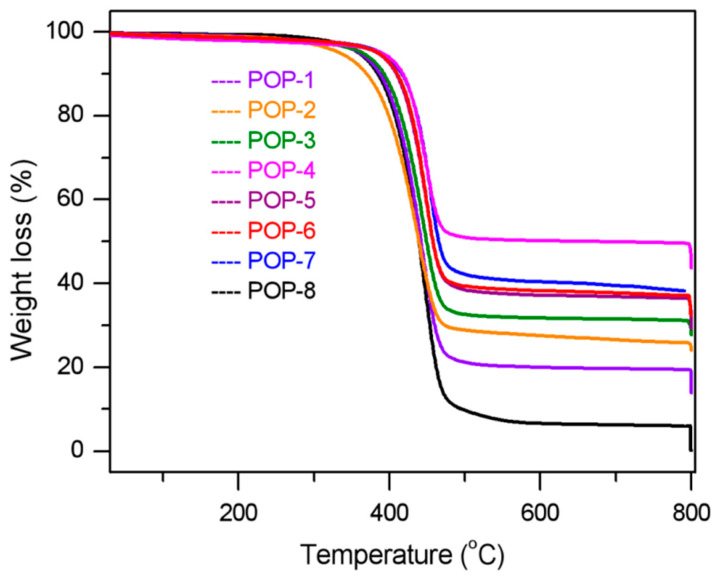
Thermogravimetric analysis (TGA) curves of the prepared TiO_2_/POP particles.

Figure 4 shows the thermogravimetric analysis (TGA) curves of the prepared TiO_2_/POP composites. The prepared TiO_2_/POP composites exhibited excellent heat stability as potential catalyst supports, and the major loss did not emerge below 300 °C. The calculated weight loss results were listed in Table 2. From Table 2, we can see that less than 3% weight loss arises under 300 °C due to slightly adsorbed monomers or solvent molecules. Take POP-8 as a reference sample without TiO_2_, the TiO_2_ content in the prepared TiO_2_/POP composites was calculated based on the weight loss value below 800 °C. The TiO_2_ content in the TiO_2_/POP composites rose from 13.8% to 44.0% when increasing the added TiO_2_ amount from 10% in POP-1 to 50% in POP-4 in the radical polymerization. The TiO_2_ content in the final product of POP-6 and POP-7 were 32.4% and 38.4%, respectively, and POP-6 and POP-7 gained better flowability than other samples.

### 3.4. XRD Analysis

**Figure 5:**
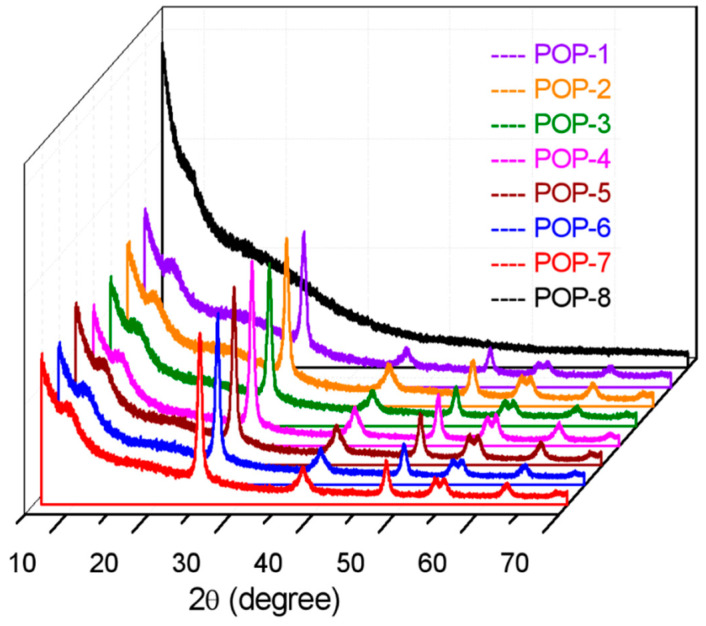
Wide angle X-ray diffraction (XRD) spectra of the prepared TiO_2_/POP particles.

Wide angle X-ray diffraction (XRD) analysis was adopted to determine the crystal type and their relative content of the nano TiO_2_ component. From Figure 5, the characteristic diffraction peaks of the anatase TiO_2_ crystal can be observed from the prepared TiO_2_/POP composites with the 2θ of 25.3°, which belongs to the (101) crystal face. In comparison, the XRD curve of the prepared POP particles (POP-9) without TiO_2_ contains no obvious diffuse peaks, indicating that the POPs prepared in the synthesis are amorphous. The relative content of TiO_2_ in the TiO_2_/POP composites could be evaluated from the value of peak height of (101) crystal face to the background peak height of around 8.8°, and the relative content of TiO_2_ is congruent to the added amount of TiO_2_ in the synthesis. Therefore, the POP-4 obtained the highest TiO_2_ amount from the XRD analysis. There are also other anatase TiO_2_ crystal faces, which could be observed in 37.8° of the (004), 48.2° of (200), and 62.8° of (204). The peaks around 27.6°, which is the characteristic peak of the rutile TiO_2_, are unobserved in these TiO_2_/POP composites.

### 3.5. Particle Size and Particle Size Distribution

**Figure 6:**
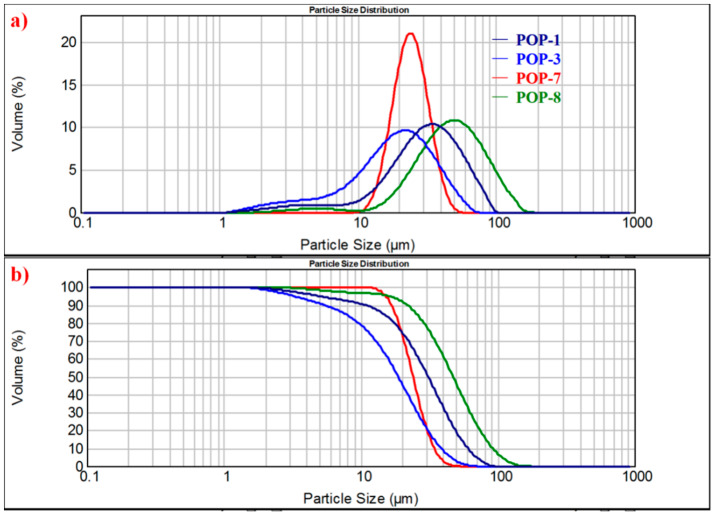
Particle size distribution curves of the prepared TiO_2_/POP particles. (**a**) Particle size distribution curves of POP-1/POP-3/POP-7/POP-8. (**b**) Cumulative curves of POP-1/POP-3/POP-7/POP-8.

Due to the replication effect during the olefin polymerization, the particle size and particle size distribution of a potential catalyst support also need to be evaluated. Generally, for a suitable olefin polymerization catalyst support in commercial applications, the particle size of D(0.5) of the support ranges from 20 to 60 μm and the particle size distribution is less than 2. The particle size and particle size distribution results of the prepared TiO_2_/POP composites are presented in Table 3 and Figure 6. 

From Table 3, we can see that relatively narrow particle size distribution with a span of less than 2 was obtained from the TiO_2_/POP composites. The average particle size D(0.5) of POP-8 was 47.3 μm, and when nano TiO_2_ beads were added, an obvious decreasing trend of particle size of the prepared TiO_2_/POP particles could be observed when increasing the TiO_2_ amount in the support synthesis. The average particle size D(0.5) reduced from 31 μm of POP-1 to 17.6 μm of POP-4. The trend could be well explained by the hydrophilic nature of the nano TiO_2_ beads absorbing the hydrophilic functional HEMA molecules, and its template role as a seed during the radical polymerization. As seen from the schematic preparation of TiO_2_/POP particles (Figure 1), the nano TiO_2_ beads (about 100 nm) could absorb HEMA, AIBN initiator, and even DVB molecules around their surfaces, and radical polymerization was initiated around the nano beads. When HEMA and DVB molecules diffuse around the nano beads, and the polymer would grow around the nano template as the copolymerization and cross-linking reaction happened. When the added nano template beads increased, more DVB/HEMA molecules polymerize around the nano beads, so narrower particle size distribution and less fine particles (1–10 μm) were noticed.

When a DVB/HEMA molar ratio of 3:1 was adopted in the synthesis of POP-6 and POP-7, narrower particle size distributions with a span of less than 1.0 were achieved, and the average particle sizes were 24.7 and 24.1 μm, respectively. From Figure 6, POP-7 obtained a normal distribution of its particle sizes with the same average particle size D(0.5) and mode values. Contrary to other samples (except POP-6 and POP-7), there exists no minor peak around 1–10 μm in POP-7, which is very common in the preparation of POP supports. The elimination of the minor peak and the acquiring of narrower normal distribution of particle size by nano TiO_2_-doping are demonstrated to be a very significant and practical approach for particle size control.

### 3.6. Surface Morphology

**Figure 7:**
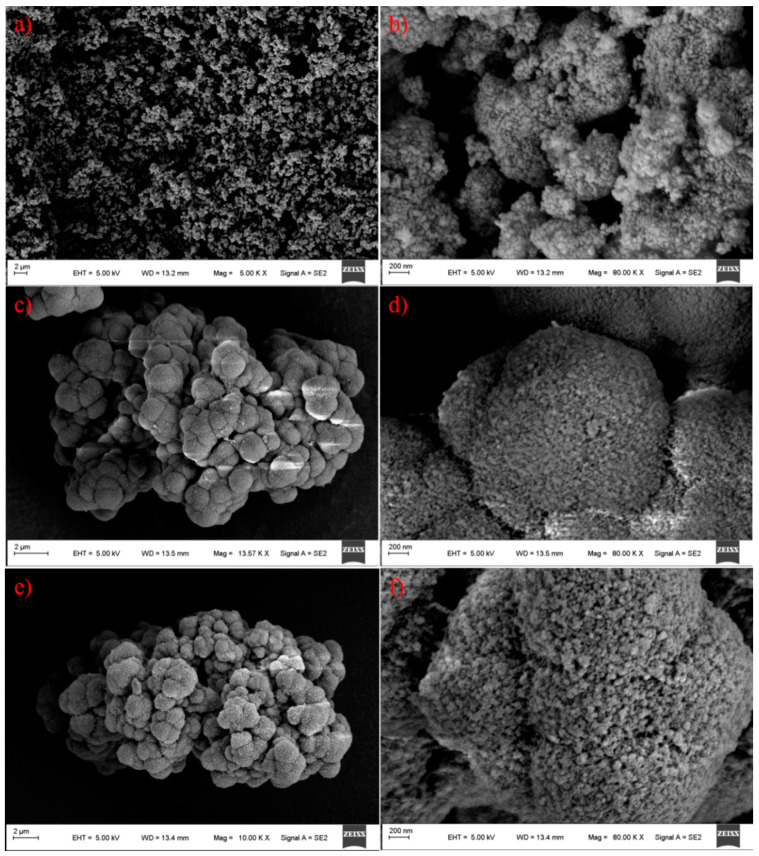
SEM images of TiO_2_ and the prepared TiO_2_/POP particles. (**a**,**b**) TiO_2_ nanoparticles; (**c**,**d**) POP-6; (**e**,**f**) POP-7.

A scanning electron microscope (SEM) was employed to examine the surface morphology of the nano-TiO_2_ beads and the prepared TiO_2_/POP particles. As illustrated in Figure 7, the TiO_2_ raw materials consists of nano aggregates, and the prepared TiO_2_/POP particles are made up of submicron spheres, which congregate to form granule units. The porous surface morphology of POP-6 and POP-7 is quite different with the TiO_2_ nano aggregates, and a great deal of nano pores in the submicron spheres could be observed. Based on the proposed particle-forming mechanism mentioned above, it is reasonable to infer that the nano TiO_2_ beads were well-dispersed in the final TiO_2_/POP particles. 

### 3.7. Ethylene Polymerization and Kinetic Curve of the Supported Metallocene Catalysts

In order to investigate the effect of the inorganic-organic supports, the metallocene complex (n-BuCp)_2_ZrCl_2_ and the MAO cocatalyst were immobilized in the prepared TiO_2_/POP composites, and then ethylene polymerization was conducted to evaluate their catalytic activity and kinetic behaviors using the produced (n-BuCp)_2_ZrCl_2_/MAO@TiO_2_/POP catalysts. From the ethylene polymerization results listed in Table 4, all four TiO_2_/POP supports supported metallocene catalysts achieved higher catalytic activity compared to the silica gel-supported commercial catalysts, indicating that the TiO_2_/POP composites could act as an excellent metallocene support in terms of ethylene polymerization. POP-7 obtained the highest catalytic activity of 4794 Kg PE/mol Zr.bar.h and productivity of 389 g PE/g cat, respectively, which is above the twice of the silica gel-supported counterpart. Moreover, POP-6 gained higher ethylene polymerization activity with low SSA (136 m^2^/g) than POP-4 with relatively higher SSA (204 m^2^/g), and we can infer that the pore structure of the support might not be the determinant factors of the catalyst polymerization activity. Apart from the Al/Zr ratio discussed in other literature and our previous work, the TiO_2_ might play an important role to influence the active sites for ethylene polymerization. The prepared TiO_2_/POP composites obtained improved activity compared to the POP support without TiO_2_ reported in our previous work, despite that the bulk densities of the prepared PE were still below that of the silica gel.

**Figure 8:**
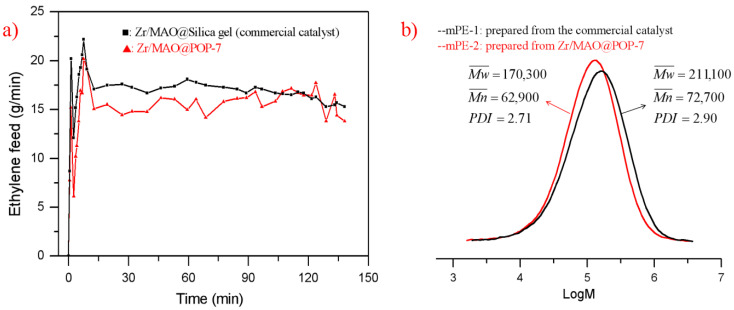
(**a**) Kinetic curves of ethylene polymerization. 0.83 g Zr/MAO@Silica gel was evaluated at 80 °C in 10 bar ethylene pressure; 0.67 g Zr/MAO@SPOP-7 was evaluated at 80 °C in 6 bar ethylene pressure. (**b**) GPC curves of two mPEs from the two catalysts.

To evaluate the ethylene polymerization kinetics, 2.0 kg hexane, 20 mL TEAL (10% wt in hexane), and 0.67g Zr/MAO@POP-7 catalyst were added into a 10 L stainless steel autoclave, and then ethylene was fed into the reactor constantly to maintain 6 bar ethylene partial pressure at 80 °C for 2 h. After the polymerization, 2142 g dry PE powder with higher ethylene polymerization activity (3197 g PE/g cat) was collected. As for the prepared TiO_2_/POP supports, the ethylene polymerization conditions seemed to play a significant role in the bulk density of the produced polymer. In the ethylene polymerization kinetic curve of Zr/MAO@POP-7 catalyst, the bulk density of the produced ethylene homo-polymer improved dramatically from 0.24 to 0.36 g/mL in higher ethylene partial pressure and polymerization activity. Significantly, the catalyst showed no obvious decay of ethylene consumption during the polymerization as illustrated in Figure 8a, and the formed Zr^+^ active sites in the Zr/MAO@POP catalyst were stable and suffered no apparent deactivation during the ethylene polymerization at about 80 °C [35]. 

As a comparison, 2283 g dry PE powder with 0.42 g/mL bulk density was obtained from 0.83 g commercial catalyst. The polymerization activity of the Zr/MAO/POP-7 catalyst (6562 kg PE/mol Zr.bar.h) was still higher than the silica gel-based metallocene catalyst (2908 kg PE/mol Zr.bar.h), partly due to a similar analogue to homogeneous polymerization, and the bulk density of mPE-2 from the Zr/MAO/POP-7 catalyst was closer to inorganic counterparts. From Figure 8b, we can see that the average molecular weight of mPE-1 (Mw = 170,300 g/mol) was less than that of mPE-2 (Mw = 211,100), and the molecular weight distribution of the mPE-2 (PDI = 2.71) was narrower than that of mPE-1 (PDI = 2.90) prepared from the silica gel-based metallocene catalyst. 

## 4. Conclusions

Free-flow Nano TiO_2_/POP particles with tunable pore structure, bulk density, and narrow pore size distribution were synthesized by precipitation polymerization with hydrophilic nano TiO_2_ beads as a template. By incorporating the inorganic nano TiO_2_ into the POPs, higher bulk density and smaller particle size of the prepared TiO_2_/POP composites could be achieved when adding a higher amount of TiO_2_. In optimalization of DVB monomer, functional HEMA comonomer and the amount of TiO_2_ added narrow pore size distribution or normal distribution curves of the prepared composites with excellent flowability and surface morphology as a catalyst support could be obtained. The ethylene polymerization evaluation showed that the prepared TiO_2_/POP composites supported metallocene catalysts obtained higher ethylene catalytic activity than the commercial counterpart. Moreover, the TiO_2_/POP composites-based metallocene catalysts exhibited excellent stable polymerization kinetics showing no obvious active sites decay during the polymerization. This inorganic-organic hybrid approach of POP synthesis would be a practical and facile way for overcoming the drawbacks of the pure organic support-based polyolefin catalysts for potential industrialization.

## Figures and Tables

**Table 1:** Pore structure from N_2_ sorption and bulk density results of nano TiO_2_/POP particles.

Entry	TiO_2_ Adding Amount/Total Monomer wt%	DVB	DVB:HEMA (Molar Ratio)	Specific Surface Area m^2^/g	Total Pore Volume cm^3^/g	Average Pore Diameter nm	Bulk Density g/cm^3^
POP-1	10%	55%	3:2	224	0.368	6.58	0.26
POP-2	20%	55%	3:2	191	0.367	7.71	0.26
POP-3	30%	55%	3:2	198	0.350	7.06	0.33
POP-4	50%	55%	3:2	204	0.312	6.12	0.35
POP-5	30%	80%	3:2	309	0.315	4.08	0.31
POP-6	37.5%	55%	3:1	136	0.150	4.41	0.25
POP-7	37.5%	80%	3:1	279	0.246	3.53	0.30
POP-8	0	80%	3:2	424	0.410	3.86	0.26

**Table 2:** The thermogravimetric analysis results of the prepared TiO_2_/POP particles.

Sample	Temperature Zone	Weight Loss, %	TiO_2_ Content (Calculated), %
POP-1	20 °C–300 °C	1.94	13.8
	20 °C–800 °C	85.98	
POP-2	20 °C–300 °C	2.51	24.0
	20 °C–800 °C	75.83	
POP-3	20 °C–300 °C	1.72	27.9
	20 °C–800 °C	71.99	
POP-4	20 °C ~300 °C	1.89	44.0
	20 °C–800 °C	55.88	
POP-5	20 °C–300 °C	1.69	29.8
	20 °C–800 °C	70.08	
POP-6	20 °C–300 °C	1.86	32.4
	20 °C–800 °C	67.44	
POP-7	20 °C–300 °C	1.46	38.4
	20 °C–800 °C	61.48	
POP-8	20 °C–300 °C	1.41	0
	20 °C–800 °C	99.79	

**Table 3:** Particle size distribution data of TiO_2_/POP particles from a Mastersizer 2000e.

Sample ^Δa^	Dv(0.1) μm	Dv(0.5) μm	Dv(0.9) μm	Mode ^Δb^ μm	Span ^Δc^
POP-1	10.8	31.0	60.7	34.9	1.61
POP-2	8.89	28.3	56.2	33.1	1.67
POP-3	5.41	18.8	38.6	22.0	1.77
POP-4	9.21	17.6	35.3	17.2	1.48
POP-5	8.10	24.3	52.2	26.7	1.82
POP-6	15.8	24.7	38.3	24.8	0.909
POP-7	16.9	24.1	34.2	24.1	0.717
POP-8	21.8	47.3	91.9	49.8	1.48

^Δ^^a^ Samples were directly detected in ethanol solvents with stirring dispersion for 150 s. ^Δ^^b^ Mode represents the peak of the particle size distribution; ^Δ^^c^ Span = [Dv(0.9) − Dv(0.1)]/Dv(0.5).

**Table 4:** Ethylene polymerization results (catalyst: (n-BuCp)_2_ZrCl_2_/MAO supported on nano TiO_2_-doped POP Particles ^e^.

Cat.	Zr (μmol/g)	Al/Zr Molar Ratio	Cat (mg)	Yield (g)	Activity (kg PE/mol.Zr.bar. h)	Productivity (g PE/g cat)	Bulk Density (g/mL)
Zr/MAO@POP-1	42.0	114	130	44.9	4112	345	0.26
Zr/MAO@POP-4	43.5	112	135	37.4	3184	277	0.22
Zr/MAO@POP-6	43.2	117	165	55.3	3879	335	0.30
Zr/MAO@POP-7	40.6	123	140	54.5	4794	389	0.24
Zr/MAO@silica *^a^	47.3	103	132	24.2	1940	183	0.36

^e^ Slurry polymerization condition: 4 Bar ethylene pressure in 800 mL reactor of stainless steel, 300 mL hexane, 80 °C, 3 mL (10 wt% in hexane) TEAL (scavenger), polymerization time: 30 min. *^a^ Silica gel-supported commercial metallocene catalyst.

## Data Availability

The data presented in this study are available in article.

## Supplementary Materials

The following are available online at https://www.mdpi.com/2079-4991/11/1/60/s1, Video S1: Highly flowable TiO_2_/POP support.
